# QbD Enabled Azacitidine Loaded Liposomal Nanoformulation and Its In Vitro Evaluation

**DOI:** 10.3390/polym13020250

**Published:** 2021-01-13

**Authors:** Prashant Kesharwani, Shadab Md, Nabil A. Alhakamy, Khaled M. Hosny, Anzarul Haque

**Affiliations:** 1 Department of Pharmaceutics, School of Pharmaceutical Education and Research, Jamia Hamdard, New Delhi 110062, India; 2 Department of Pharmaceutics, Faculty of Pharmacy, King Abdulaziz University, Jeddah 21589, Saudi Arabia; shaque@kau.edu.sa (S.M.); nalhakamy@kau.edu.sa (N.A.A.); elswaify2000@yahoo.com (K.M.H.); 3 Center of Excellence for Drug Research & Pharmaceutical Industries, King Abdulaziz University, Jeddah 21589, Saudi Arabia; 4 Department of Pharmacognosy, College of Pharmacy, Prince Sattam bin Abdulaziz University, Al-kharj 16278, Saudi Arabia; a.anwarulhaque@psau.edu.sa

**Keywords:** liposomes, box-behnken design, azacitidine, lipid film hydration technique, breast cancer, MCF-7 cell line, chemotherapy

## Abstract

Azacitidine (AZA), an inhibitor of DNA methyltransferase, is a commonly recognized drug used in clinical treatment for myelodysplastic syndrome and breast cancer. Due to higher aqueous solubility and negative log P of AZA causes poor cancer cell permeation and controlled release. The objective of the present study was to formulate and optimize AZA-loaded liposome (AZA-LIPO) for breast cancer chemotherapy by using Box Behnken design (BBD) and in vitro evaluation using MCF-7 cells. AZA-LIPO were prepared using a thin film hydration technique and characterization study was performed by using FTIR and DSC. The prepared formulations were optimized using BBD and the optimized formulation was further subjected for particle size, surface charges, polydispersity index (PDI), drug loading, entrapment efficiency, TEM, XRD, in-vitro drug release and hemolytic toxicity. The mean particle size of optimized AZA-LIPO was 127 nm. Entrapment efficiency and drug loading of AZA-LIPO was found to be 85.2% ± 0.5 and 6.82 ± 1.6%, respectively. Further, in vitro drug release study showed preliminary burst release in 2 h followed by a sustained release for 36 h in phosphate buffer at different pH (4.0, 5.5, and 7.4) as compared to free drug. Drug release was found to be pH dependent, as the pH was increased, the drug release rate was found to be low. Time-dependent cell viability assay exhibited significant higher cell viability and higher internalization than free AZA in MCF-7 cells. AZA-LIPO were more effective than the free AZA in reducing Bcl2 expression, while increasing pro-apoptotic Bax and caspase-3 activity. The result showed that the formulated biocompatible AZA-LIPO nano-formulations may be used as an efficient anti-cancer drug delivery system for the treatment of breast cancer after establishing preclinical and clinical studies.

## 1. Introduction

In 1960s Azacitidine (AZA) was primarily synthesized as an analogue of 2′-deoxycitidine a DNA methyl transferase inhibitor [1,2]. AZA is the most preferred and recommended drug for myelodysplastic syndrome (MDS) and breast cancer treatment [3,4,5]. However, the clinical limitation of AZA drug is due to the variations in their physicochemical properties such as very high aqueous solubility (22.67 mg/mL), high metabolism, low half-life (less than 4 h), low oral bioavailability and poor stability profile under neutral pH for 7 days at 4 °C [6]. Due to high AZA hydrophilicity, which results in a failure of the tailor-made profile and leads to non-specific systemic distribution of AZA along with low drug permeation at the cancer site [7]. Whereas at higher dose it slows down the cell propagation by forming covalent linkage with DNA methyltransferase enzyme which inhibit AZA synthesis [8]. Liposomes are the nanocarrier, which are small spherical bilayered lipid vesicles, hence allowing the incorporation of both hydrophilic and lipophilic material in their respective compartments [9,10]. Liposomes are promising carriers for delivery of higher drug payloads to target site and prolongs the circulation time in systemic circulation [11,12]. Liposomes have biocompatible and biodegradable properties and most acceptable as drug delivery vehicles. These nanocarriers can move through lipid bilayers of cancer cells with increased AZA concentration at the site of action and eventually increase cytotoxicity [13,14]. To the best of the authors’ knowledge, to date, AZA-loaded liposomes were not formulated. The advantage of using the formulation of AZA-loaded liposomes is that it will provide easy permeation in cells and further provide controlled release of AZA at the site of cancer. Therefore, AZA-encapsulated liposomes (AZA-LIPO) with an approach for targeting nanoparticles as chemotherapeutic agents using soy lecithin and cholesterol and a thin-film hydration technique was prepared. Soya lecithin and cholesterol are excipients were used for preparation of AZA loaded liposomal formulation. Both soya lecithin and cholesterol are approved by the United States Food and Drug Administration (USFDA) and are enlisted as generally regarded as safe (GRAS) [15]. Quality by design (QbD) approaches were implemented during the development of AZA-LIPO to evaluate the effects of various parameters related to process aspects on product development [16]. QbD is a risk assessment-based and systematic approach for improving the quality and should be applied during the preliminary stage of formulation optimization, designing and development phase for qualitative uniformity in products. This approach also helps in analyzing the consequences of critical process parameter (CPP) on critical quality attributes (CQA) [17]. The Box Behnken Design (BBD) is implemented to attain the quality target product profile (QTPP) in the minimum number of experimental runs [18]. The effects of major CPP on the CQA % entrapment efficiency and particle size were studied using BBD [19]. Furthermore, the optimized AZA loaded liposomes were characterized using Fourier transform infrared spectroscopy (FTIR), differential scanning calorimetry, transmission electron microscopy, x-ray diffraction study and in vitro release study. Cellular uptake study, cell viability, Bax, Bcl-2 and caspase activity of optimized AZA loaded liposomes and free AZA were investigated in mcf-7 breast cancer cells.

## 2. Materials and Methods

### 2.1. Chemicals

AZA was procured as a gift sample from Micro Labs Pvt. Ltd. (Bangalore, Karnataka, India). Lecithin, cholesterol and 3-(4, 5-Dimethylthiazol-2-yl)-2, 5-diphenyltetrazolium bromide) (MTT) was purchased from HiMedia laboratories Pvt. Ltd. (Mumbai, India). Chloroform was purchased from Lobachemie Pvt Ltd. (Mumbai (India) and deionized water was purchased from Milli-Q system (Millipore, Bedford, Mumbai, India). Fluorescein isothiocynate (FITC) and 4′,6-diamidino-2-phenylindole (DAPI) were purchased from Sigma Aldrich (Mumbai, India). All other solvents and chemicals were of analytical grade and obtained from other local supplier.

### 2.2. Preparation Method of AZA Loaded Liposomes

**Figure 1:**
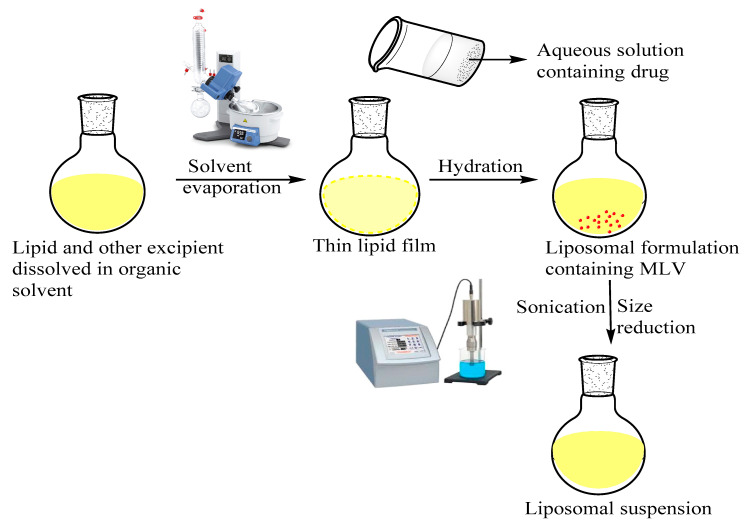
Graphical representation for the preparation of Azacitidine (AZA)-loaded liposomes.

The AZA encapsulated liposomes (AZA-LIPO) were prepared by lipid thin film hydration technique. The AZA-LIPO comprises of cholesterol (CH) and lecithin (PC) in the ratio of (3:7) Soya CH/PC (in given quantity) were dissolved in 100 mL round bottom flask (RBF) containing 5 mL chloroform. The organic solvent was evaporated using Rota vapour (IKA RV10) at speed of 65 rpm and temperature of 37 °C for 10 min to obtained thin lipid film. Excessive solvents were removed by keeping RBF (Round bottom flask) under vacuum for 24 h. The drug was dissolved in phosphate buffer pH 7.4 (10 mL) for hydration of lipid thin film slowly for 30 min until it get completely dissolved [20]. Multilamellar vesicular liposomes formed were probe sonicated for 15 min at amplitude of 20 W to obtained unilamellar vesicular liposomal suspension [21]. The liposomal suspension was further subjected to freeze drying by using 5% *w*/*v* of mannitol to obtained lyophilized powder and stored at 4 °C in airtight container for further experiments [22]. Graphical representation for the preparation of AZA loaded liposomes has been represented in the Figure 1.

### 2.3. Box Behnken Design and Optimization Condition (BBD)

In this design BBD includes multiple center points with three independent factors and 3 levels with 17 experimental runs were utilized to assemble second order polynomial equation to optimize batch with desirable characteristics [23]. This design was implemented to evaluate simultaneous effect of CPP on responses using Design Expert Software (Version 7.16, Stat-Ease, Inc., Minneapolis, MN, USA). The crucial CPP were *w*/*v* lipid concentration (Y_1_), *w*/*v* cholesterol concentration (Y_2_) and sonication time (min) (Y_3_) with their respective levels −1(low), 0 (medium) and +1 (high) whereas the CQA were particle size (nm) and 3% drug entrapment efficiency (%) as shown in Table 1. The quadratic equations obtained from BBD is as follows:

X = B_0_ + B_1_Y_1_ + B_2_Y_2_ + B_3_Y_3_ + B_12_Y_1_Y_2_ + B_13_Y_1_Y_3_ + B_23_Y_2_Y_3_ + B_11_Y_1_^2^ + B_22_Y_2_^2^ + B_33_Y_3_^2^ (1)

where, X is CQA (responses), B_0_ is denoted as intercept, B_1_ to B_33_ in equation is regression coefficients of given factors and Y_1_ to Y_3_ are the code given to independent factors. Considerable effect of each independent variable with their respective levels towards the dependent variable were predicted via analysis of variance (ANOVA) and *p* value < 0.05 [24]. The check points were evaluated by batch T1 and T2 to predict the independent factor on responses of desired batch. The response surface model was generated to observe the effect of CQA on AZA-LIPO particle size and % EE.

### 2.4. Particle Size, PDI and Zeta Potential

The particle size, polydispersity index (PDI), and zeta potential were determined using Quasi elastic light scattering techniques. AZA-loaded liposomal suspension was diluted with 1:100 distilled water and analyzed by means Nano-ZS zetasizer (Malvern instruments, Malvern, UK) [25,26,27].

### 2.5. Drug Entrapment and Drug Loading Efficiency

The drug content of AZA –LIPO was estimated by high speed centrifugation method. Prepared AZA-LIPO suspensions (1 mL) were subjected for centrifugation on laboratory centrifuge (sigma 2–16PK Labmate (Asia) Pvt. Ltd.) at 12,000 rpm for the time period of 40 min [28]. The supernatant was taken and preceded for further analysis in UV−VIS spectrophotometer (LABINDIA UV 3000) at the wavelength of 242 nm for the analysis of un-entrapped AZA in the liposomal formulation and the percent drug entrapment efficiency was calculated [29]. The percentage Drug loading (%DL) was calculated from the pellet part of centrifuged AZA-LIPO suspension.

(2) $$\%\mathrm{EE}=\frac{\mathrm{amount}\;\mathrm{of}\;\mathrm{AZA}\;\mathrm{added}\;\mathrm{in}\;\mathrm{liposome}-\mathrm{amount}\;\mathrm{of}\;\mathrm{unentrapped}\;\mathrm{AZA}}{\mathrm{amount}\;\mathrm{of}\;\mathrm{AZA}\;\mathrm{added}\;\mathrm{in}\;\mathrm{liposome}\;}\;\times \;100$$

(3) $$\%\mathrm{DL}=\frac{\mathrm{amount}\;\mathrm{of}\;\mathrm{AZA}\;\mathrm{added}\;\mathrm{in}\;\mathrm{liposome}-\mathrm{amount}\;\mathrm{of}\;\mathrm{unentrappedAZA}}{\mathrm{total}\;\mathrm{amount}\;\mathrm{of}\;\mathrm{liposomal}\;\mathrm{formulation}\;\left(\mathrm{drug}+\mathrm{excipients}\right)}\;\times \;100$$

### 2.6. Fourier Transform Infrared Spectroscopy (FTIR)

AZA, Lyophilized AZA –LIPO and physical mixtures of excipients (Cholesterol, soy lecithin, mannitol) samples were examined under FTIR spectrophotometer. The sample was converted into KBr pellets using a hydraulic press. Then, pellets were exposed to infrared radiation to pass through samples [30,31]. The spectrums were recorded in the region of 4000 to 400 cm^−1^.

### 2.7. Differential Scanning Calorimetry (DSC)

Thermo analytical study was performed by a DSC instrument (TA Instrument Trios v4 2.13662 software) to determine the physical state of the drug, mannitol and lyophilized AZA-LIPO. The study was conceded at the temperature range between 50 and 250 °C at a scanning rate of 10 °C min^−1^. The samples for analysis were prepared by weighing accurate quantity of Drug (AZA), mannitol and AZA-LIPO formulation in aluminum pan and then crimped with lid. The crimped pan containing sample was kept in DSC instruments with reference blank pan at the back to analyze the sample [32]. DSC thermogram of samples (AZA, mannitol and AZA-LIPO formulation) was recorded [33].

### 2.8. X-Ray Diffraction Study

The samples were analyzed on by X-ray powder diffractometer (XRD-2000, Rigaku, Tokyo, Japan) by using Cu-K α radiation set with nickel filter at 1.5404 angstrom in order to evaluate the X-ray powder diffraction pattern of pure AZA, excipients and AZA-LIPO lyophilized powder. The samples were putted down on a glass sample holder and the diffraction patterns were recorded [34,35].

### 2.9. Transmission Electron Microscopy (TEM)

AZA-LIPO morphology were imaged on TEM. The liposomes were stained with 2% uranyl acetate, formulation is placed on carbon coated grids of 400 mesh and kept on room temperature for 60 sec. Liposomes were observed under the microscope with accelerating voltage of 60–100 kV [36,37].

### 2.10. In-Vitro Drug Release Study

The in vitro release study of free AZA (5 mg) and dose equivalent to AZA liposomal suspension was performed at Phosphate buffer saline (PBS) at different pH (4.0, 5.5, and 7.4). Free AZA nanosuspension was prepared by dissolving the 5 mg of AZA (amount of AZA added in formulation per mL) in 1 mL of PBS (pH 7.4) followed by sonication [38]. The drug release experiment was carried out in dialysis bag (nitrocellulose membrane, Mol. Wt. cut off 12,000 Da). Dialysis bag was activated by treatment with phosphate buffer solution half an hour prior to release study. Equivalent amount of free drug nanosuspension and AZA-LIPO nanosuspension were placed in nitrocellulose bags and the ends of bag were sealed by tie thread. PBS at different pH (4.0, 5.5, and 7.4) is used as dissolution medium for release study [39,40]. The nitrocellulose bags containing formulation were kept in 100 mL of dissolution medium at 37 °C at 100 rpm over a magnetic stirrer for 24 h. Samples were withdrawn at different time intervals and the sink condition was maintained by replenishing the equal amount of PBS. The absorbance was taken on a UV-Visible spectrophotometer at 243 nm. A drug release study was conducted in triplicates and the drug release kinetic model was applied to obtained release data.

### 2.11. Hemolytic Toxicity

Hemolytic toxicity was examined by hemoglobin content in the supernatant of the centrifuged RBC suspension spectrophotometrically followed by our previously reported method [31,40,41,42,43]. RBCs were collected from whole human blood by centrifugation, followed by RBCs washing with normal saline to obtain a clear colorless supernatant. Cells were suspended in normal saline and developed formulations were added at different concentration and tubes could stand for half an hour with gentle intermittent shaking and were centrifuged for 15 min at 3000 rpm. The supernatants were pooled, and spectrophotometric analysis was done at 540 nm to determine the degree of hemolysis.

### 2.12. Cytotoxicity Assay

The cytotoxicity assay of Free AZA, blank liposome and AZA-LIPO on MCF-7 breast cancer cells were done by MTT 3-(4,5-dimethylthiazol-2-yl)- 2,5-diphenyltetrazolium assay. In a 96 well microtiter plate MCF-7 cells (2 × 10^4^/well) were seeded and left to attach for 24 h. Fresh prepared media was added and the cells were treated different concentrations of free AZA, blank liposome and AZA-LIPO (5, 10 and 15 µM AZA/well) culture medium treated group is considered as control, incubated at 37 °C for 24 h in CO_2_ incubator [44]. The cells were rinsed with PBS 7.4 and treated with MTT reagent (5 mg/mL) and subjected to incubate for 4 h [45,46]. The formazan crystals were dissolve via adding 100 µL of DMSO in a 96 well plate the generated by living cells. Then the absorbance was observed by microplate reader at a same excitation and emission wavelength of 570 nm. PBS-treated cells function as controls for the formulation-treated cells, and considered to be 100% viable [47]. Data were shown as a mean ± standard deviation (SD), and the extent of cytotoxicity was observed for 24 and 48 h after treatment.

(4) $$\%\mathrm{Cell}\;\mathrm{viability}=\frac{\left(\mathrm{Absorbance}\;\mathrm{treatment}\right)}{\left(\mathrm{Control}\;\mathrm{absorbance}\right)}*100$$

### 2.13. Confocal Microscopy

To evaluate the cell internalization efficiency of AZA-LIPO in MCF-7 cells confocal microscopy studies were conducted. MCF-7 cells were incubated at a density of 1 × 10^3^ cells in six well poly-l-lysine coated plate and incubated for 24 h. After incubation cells are treated with FITC loaded Liposomes (FITC-LIPO (20 µL) and incubated for 4 h. Further, cells were treated with DAPI and incubated for 15 min. Cell uptake images were taken by a Confocal Laser Scanning Microscope [45,48].

### 2.14. Determination of Bax, Bcl-2 and Caspase-3 Proteins Expression

Analysis of Bax, Bcl-2 and caspase-3 protein was performed using the ELISA method. The MCF-7 cells (2 × 10^4^/well) were seeded into 96-well plate. The cells were treated with free AZA, AZA-LPO and blank liposome samples for 24 h. Thereafter, the wells were incubated with monoclonal antibodies against Bax (Ab-1) (DRG^®^ Human Bax ELISA Kit), Bcl-2 (Zymed^®^ Bcl-2 ELISA Kit) and caspase-3 (Invitrogen ELISA kit). The procedure was followed as per manufacturer instruction [49].

### 2.15. Statistical Analysis

The experimental design and statistical calculations were analyzed in design expert software. In BBD 17 runs with 3 center points were implemented to validate polynomial equation by ANOVA (Analysis of variance).

## 3. Result and Discussion

### 3.1. Preparation of AZA-LIPO

The lipid thin film hydration method was used to load the AZA a hydrophilic drug into the liposome. Liposomal formulation can entrap the hydrophilic drugs in the outer layer of vesicle. Formulated liposomal nanosuspensions were sonicated and at last liposomes were freeze-dried and stored at 4 °C using 5% *w*/*v* mannitol as a cryoprotectant.

### 3.2. Optimization of AZA-LIPO

The lipid film hydration technique is a widely preferred method for the preparation of liposomes, even though it requires optimization for process parameters with preferred particle size and % entrapment efficiency. Process parameters such as lipid weight concentration (mg), cholesterol weight concentration (mg) and sonication time (min) affect the liposome characteristics. Some other parameters such as rota vapor speed (rpm), film hydration time (min) also showed effect on liposome characteristics. Effects of various independent variables on responses were shown in Table 2.

The parameters for liposome selection were set with low particle size (100–250 nm) and higher entrapment efficiency as needed. The liposome formulation with its expected data from DOE software and the observed data from experimental data were found to be closer. Two check points T1 and T2 were utilized for the validation of actual and predicted values as shown in Table 3. The *p* < 0.05 values of responses such as % EE and particle size were found to be significant.

### 3.3. Particle Size

The AZA-LIPO film was formed by dissolved drug hydrating the thin film of lipid organic phase containing lipid and cholesterol. The addition of cholesterol stabilizes the phospholipid bilayer of lecithin, while simultaneously reducing the size of multilamellar vesicles by probe sonication to obtain unilamellar vesicular liposome. The change of multilamellar to unilamellar vesicles significantly affects particle size and PDI of liposomal formulation. Mean particle size ranges from 104 to 235 nm and was prejudiced by lecithin concentration, cholesterol concentration and sonication time. The response surface plots were calculated by using polynomial regression equation.

Particle size = 190.40 + 1.13A + 14.63B − 33.75C + 2.50AB − 4.75AC + 20.25BC − 64.45A^2^ −14.45B^2^ + 14.80C^2^ (5)

**Figure 2:**
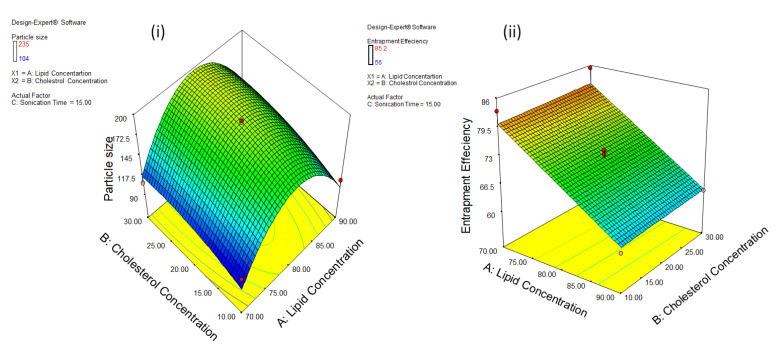
(**i**) Response surface plot and (**ii**) contour plot for particle size and entrapment efficiency.

The correlation coefficient (R^2^) and adjusted R^2^ values for particle size was analyzed to be 0.95 and 0.89. The particle size of AZA-LIPO increases with increase in concentration of lecithin and sonication time with higher cholesterol shows inverse relationship. The response surface plot for particle size is shown in Figure 2. The lack of fit value *p* > 0.005 was found to be significant in the ANOVA model for particle size. Liposome with particle size <200 nm are efficient to pass the cell membrane and to release the drug in cytoplasm.

### 3.4. Entrapment Efficiency

Factors which effect the entrapment efficiency which is important for the encapsulation of hydrophilic drug into liposomal formulation. The design applied was found significant (*p* > 0.0004) and the values for correlation coefficient R^2^ and adjusted R^2^ was found to be 0.88 and 0.80. The percentage entrapment efficiency of AZA-LIPO was found to be in the range 56% to 85%. The polynomial equation obtained by design for percent entrapment efficiency is as follows:

%EE = 71.14 − 8.82A + 0.56B + 2.09C + 0.001AB + 3.00AC − 5.33BC(6)

The polynomial equation showed that lipid concentration and sonication time are the primary parameters that affects entrapment efficiency. Lipid concentration and sonication time is directly proportional to % entrapment efficiency. The response surface plot for entrapment efficiency is shown in Figure 2. The lack of fit value was found to be significant in the ANOVA model for % EE indicating that the regression equation was well fitted. Hydrophilic cavity between bilayers of liposome enhances the encapsulation capacity of hydrophilic drugs. We also found that with the increase in particles size of formulations, the drug loading and entrapment efficiency has also been increased at certain points; after that, increase in particle size leads to decrease in drug loading and entrapment efficiency due to less soluble formulation. This has not always happened, there are various factors that can affect particle size, entrapment and loading together and individually. Our results are well in accordance with the previous studies [27].

### 3.5. Data Optimization and Model Validation

**Figure 3:**
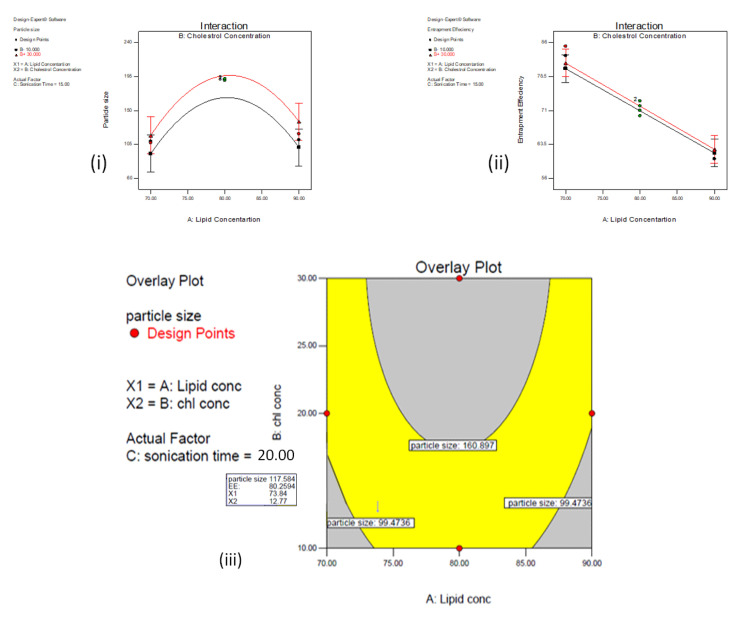
Interaction plot for (**i**) particle size, (**ii**) entrapment efficiency and (**iii**) overlay plot.

**Figure 4:**
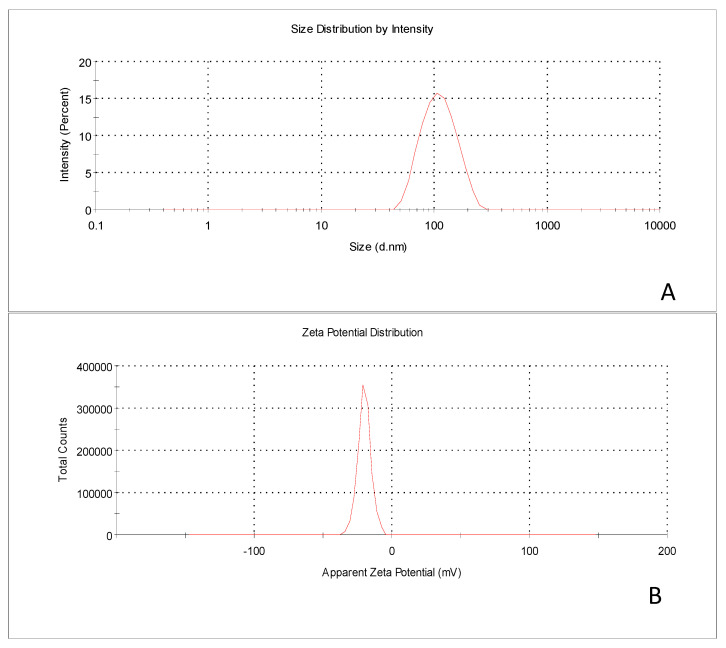
(**A**) Particle size distribution and (**B**) zeta Potential of AZA-LIPO formulation.

The design expert software 7.0 generates over lay plot contain two regions shown in the yellow and gray region. The yellow region showed a design feasible area and the gray region indicates that the particular region is not suitable to get an optimized batch. The desirability and overlay plot were based on optimized process attributes such as lipid concentration 73.84 mg, cholesterol concentration 12.77 mg and sonication time 20 min. The predicted values for responses such as particle size 117.58 nm and entrapment efficiency 80.25% were obtained from overlay plot and the desirability criteria for this model is 0.978 as shown in Figure 3. The particle size, PDI and zeta potential of optimized batch 7:3:1 of AZA-LIPO was found to be 107 ± 1.1 nm (Figure 4A), 0.035 ± 0.5 and −25 mV respectively (Figure 4B) indicates high storage stability of liposome. The % EE and %DL of optimized batch was found to be 85.2 ± 0.5% and 5.82 ± 2% as shown in Table 4**.**

### 3.6. FTIR

**Figure 5:**
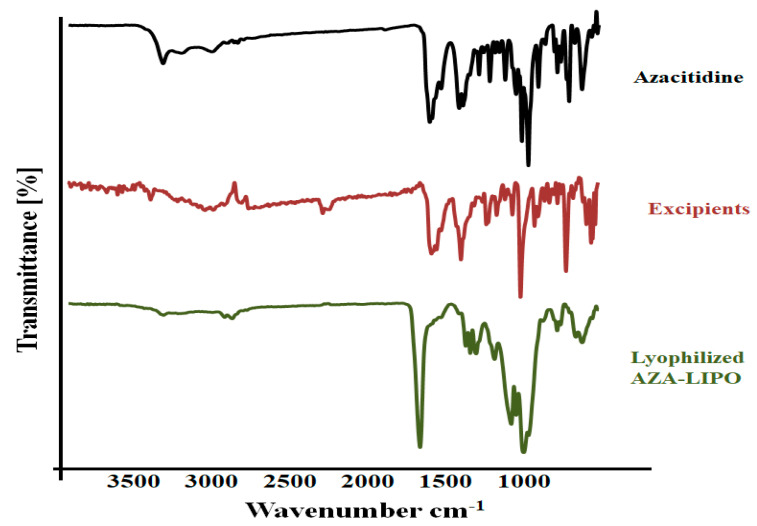
FTIR spectra of AZA, excipients and AZA-LIPO formulation.

The FTIR spectra of AZA, excipients and AZA-LIPO were analyzed as shown in Figure 5. FTIR spectra of AZA show a characteristic peak at 1528.5 cm^−1^ which indicates the presence of amine group (N-H) and a sharp peak at 2351.1 cm^−1^ clears the presence of OH group. The FTIR spectra of excipients (lecithin + cholesterol) exhibit characteristics peak at 1402.1 cm^−1^ and 1694.91 cm^−1^. However, the lyophilized AZA-LIPO formulation exhibit smooth spectra with single characteristics sharp peak at 1636.81 cm^−1^ which indicates that the drug is incorporated into the formulation and no interactions were taking place between drugs and lipids. The lyophilized formulation showed that most of the peaks have vanished, it suggests that certain physical interactions such as formation of hydrogen bonds have occurred between functional groups present in drugs and lipids, but no chemical interactions occur [40].

### 3.7. DSC

**Figure 6:**
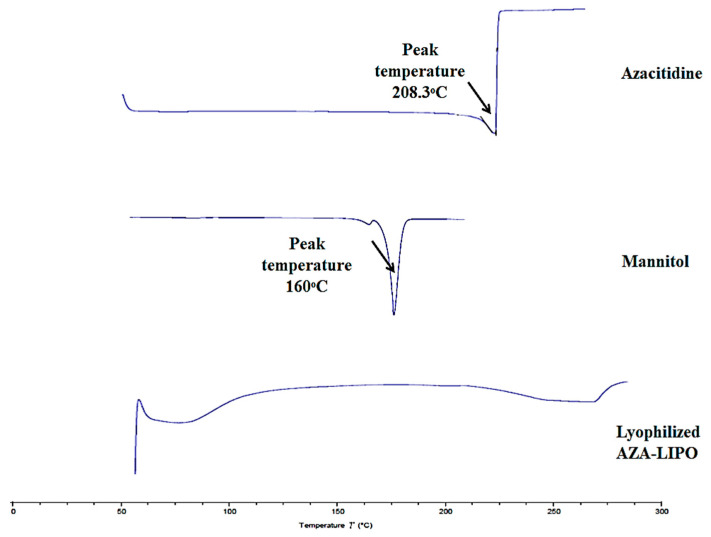
Comparative DSC thermo gram of AZA, excipients and AZA-LIPO formulation.

DSC thermogram of AZA, physical mixture (lecithin + cholesterol+ AZA+ mannitol) and AZA-LIPO were shown in Figure 6. AZA exhibits a sharp endothermic peak at 208.3 °C which represents the crystalline nature of drugs. The DSC mannitol endothermic thermogram was found at 160 °C. However, the DSC endothermic thermogram for AZA-LIPO showed a diminished peak, which represented complete entrapment of AZA in liposome and conversion of crystalline to an amorphous form.

### 3.8. X-Ray Diffraction Study

**Figure 7:**
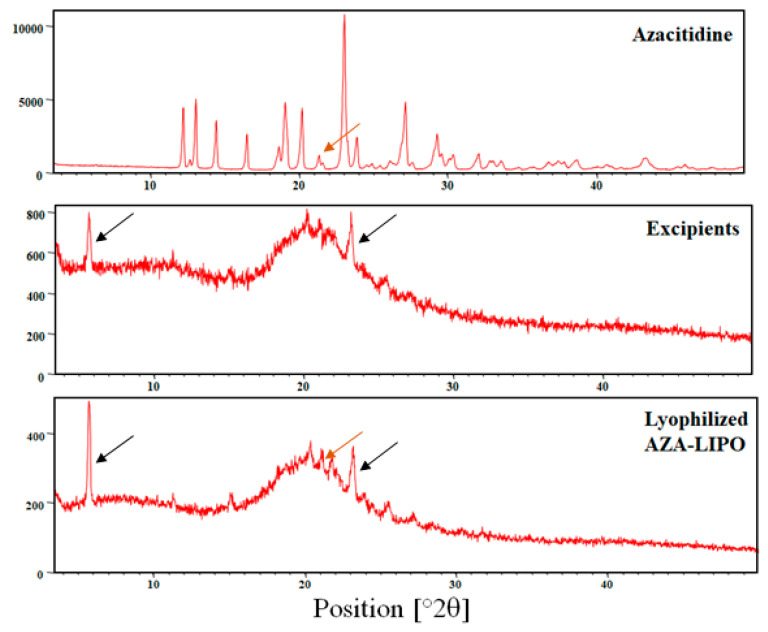
XRD of AZA, excipients and AZA-LIPO formulation.

X-ray diffraction study of pure AZA, excipients (Soya lecithin, cholesterol and mannitol) and AZA-LIPO were shown in Figure 7. XRD data show a sharp peak for pure AZA which indicates the crystalline nature of drug; whereas, sharp peaks were also observed in case of excipients. AZA-LIPO showed broad peaks, and sharp diffraction peaks were not observed. This may be due to the entrapment of AZA in liposomes. Some sharp peaks appeared in AZA-LIPO formulation could be due to presence of mannitol in formulation.

### 3.9. TEM

**Figure 8:**
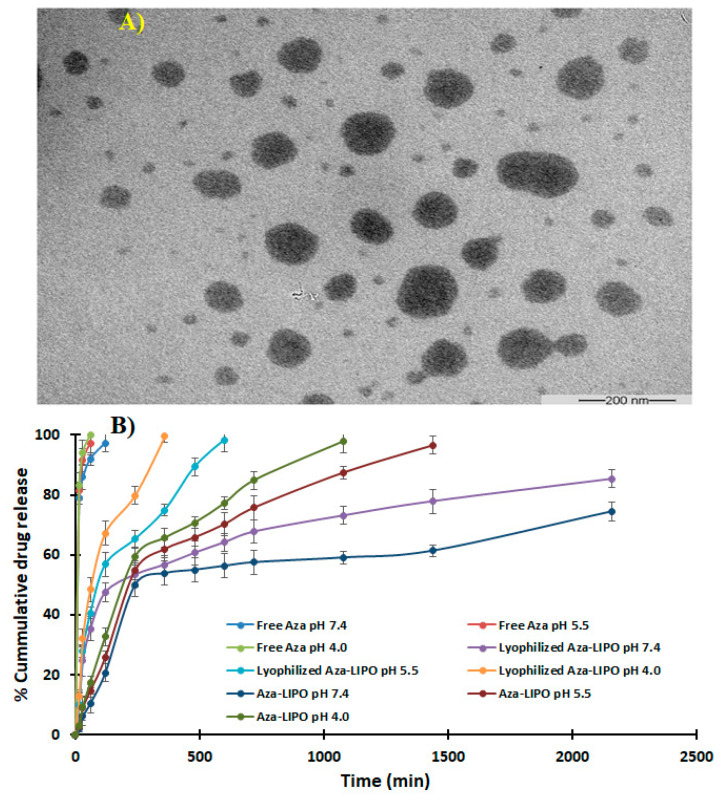
(**A**) TEM image of AZA-LIPO formulation; (**B**) in vitro drug release profile of developed formulations (n = 6).

TEM images of AZA-LIPO show smooth spherical vesicles. TEM imaged the liposome internal morphology and displayed particle size in a nanometre range (Figure 8A). Liposome size was less than 200 nm suitable for enhanced permeation and retention effect. The zetasizer data confirmed that polydispersity index (PDI) is in the range 0.1–0.2 which showed homogenous size distribution which is good for tumor targeting.

### 3.10. In Vitro Drug Release Study

The release study of free AZA 5 mg/mL and equal amount of AZA-LIPO nano suspension and lyophilized AZA-LIPO liposomes was performed at different pH (4.0, 5.5, and 7.4) to calculate the release rate of drug as shown in Figure 8B. For free AZA dissolved in water, it was observed that more than 90% drug release was taken place within 2 h (91.97 ± 2.2% at pH 7.4, 97.23 ± 4.3% at pH 5.5 and 99.98 ± 3.1% at pH 4.0). However, lyophilized AZA-LIPO showed a sustained drug release pattern at all the experimental pHs. The AZA-LIPO exhibited only 74 ± 2.3% of drug release in comparison to lyophilized formulation at pH 7.4. Higher release of AZA in freeze-dried formulation was observed due to conversion of the drug into amorphous form. However, AZA is hydrophilic in nature, and the release of drug release was prolonged to 36 h due to liposomal formulation. Drug release was found to be pH dependent, and drug release was comparatively faster at low tumor pH (pH 4.0) which is advantageous for us as an anticancer candidate. As the pH was increased, the drug release rate was found to be low. Our results are well in accordance with the previous studies [31,37,41,50,51,52,53,54]. The drug release obtained from AZA-LIPO followed a mixed order release due to burst release initially followed by sustained release pattern for freeze dried formulation, which prolonged the release of drug from liposome bilayers.

### 3.11. Hemolytic Toxicity

**Figure 9:**
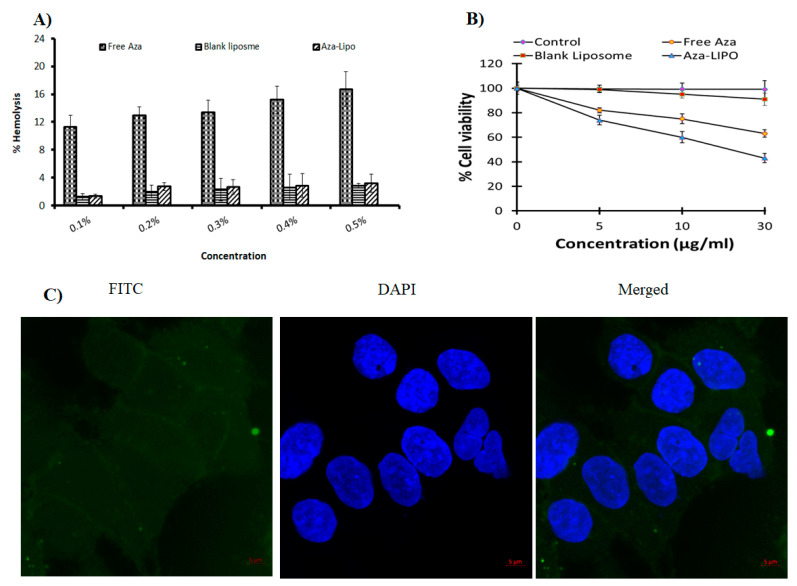
(**A**) Hemolysis toxicity profile (n = 6), (**B**) cytotoxicity assay (n = 6) and (**C**) confocal microscopy of the developed formulations.

The hemolytic toxicity was estimated in terms of percent RBC hemolysis. At highest experimental concentration (0.5%) it was observed that free drug Aza exhibited 16.74 ± 2.48%, while blank liposomes and Aza-Lipo showed 2.86 ± 0.34% and 3.16 ± 1.32% RBC hemolysis, respectively. Liposome-based formulation significantly decreased RBC hemolysis due to Aza’s shielding/locking within the liposome. Our observations clearly confirmed the biocompatibility of formed liposome formulations and may be useful in future as a promising drug delivery alternative (Figure 9A).

### 3.12. Cytotoxicity Assay

An MTT assay was performed to determine in vitro cytotoxicity potential of free AZA, blank liposome and AZA- LIPO. The inhibitory concentration value of AZA and AZA-LIPO on MCF-7 cell line was found to be 39.54 µM and 23.23 µM respectively (Figure 9B). Free AZA and formulations were showing a dose-dependent reduction of cell viability on MCF-7 cell line. The blank liposome showed lowest cytotoxicity at highest drug equivalent concentration of 30 µM. The viability of cells treated with AZA-LIPO 30 µM concentration found to be 43 ± 2.3% in comparison to free AZA 63 ± 4.4% at a drug equivalent concentration at 36 h. The dose 30 µM of AZA-LIPO showed a significant increase (*p* < 0.05) in cytotoxicity in comparison to free AZA. Results revealed that particle size less than 200 nm and controlled release of optimized formulation are efficient in killing the cancer cells. The higher cell uptake/permeability of AZA-LIPO in cancer cells also enhanced the formulation cytotoxicity. Our results were well in agreement with the reported literature where Kashyap and co-worker have found similar results using PLGA as a nanocarrier [2].

### 3.13. Confocal Microscopy

The cellular uptake study was conducted to determine the cellular internalization of AZA-LIPO in MCF-7 cell. Due to negative log *p* value of AZA, it reduces the cellular uptake in cancer cells. FITC used which mimics as AZA in FITC-LIPO to evaluate the uptake of AZA in cells. FITC-LIPO treated cells exhibited green color FITC fluorescence in the cytoplasm of cells. Nano-sized AZA-LIPO showed comparatively slightly better cell uptake and enhanced cytotoxicity in MCF-7 cells (Figure 9C). Our results were in accordance with the recent report of Kashyap et al., 2020 where they used same drug but using different carrier i.e., PLGA nanoparticle [2].

### 3.14. Effect of the AZA LPO on the Expression of Bax, Bcl-2 and Caspase-3 Proteins

**Figure 10:**
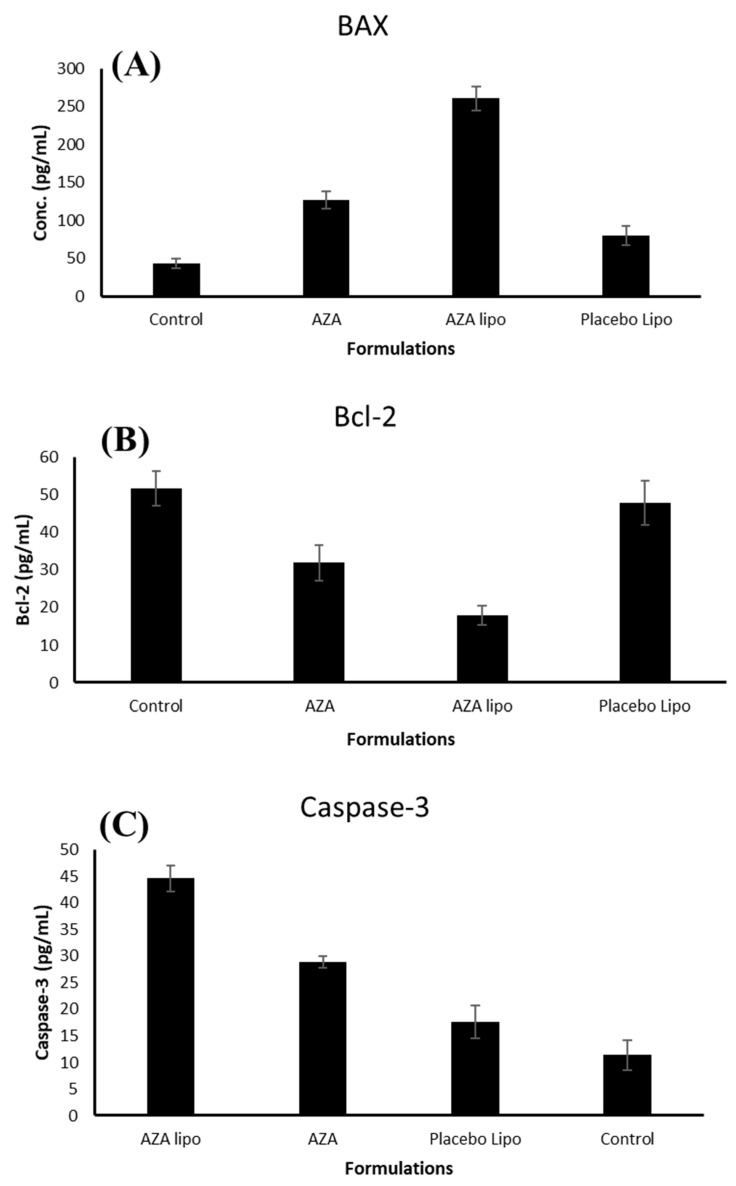
Effect of the IC50 concentrations of fee AZA, placebo liposomes and AZA-Lipo on the expression of Bax, Bcl-2 protein and caspase-3 activity in A549 cancer cells (*p* < 0.05 for free AZA vs. AZA Lipo formulation.

Higher Bcl-2 expression increases antiapoptotic activity, while Bax and caspase-3 expression induces apoptosis [50] (Figure 10A). AZA-LPO treatment produced a marked decrease in Bcl-2 expression relative to control and free AZA. On the other hand, treatment with AZA-LPO produced a significant (*p* < 0.05) increase in the expression of Bax (260 ± 15.79 pg/mL) compared with free AZA (126.7 ± 11.13 pg/mL) (Figure 10B). Caspase-3 activity is associated with cancer cell apoptosis [55,56]. Treatment with AZA-LPO (44.68 ± 2.45) produced a significant (*p* < 0.05) increase in caspase-3 activity compared to free AZA (28.89) (Figure 10C). Increased liposome efficacy relative to free AZA may be due to improved targeting at the cancer site. Another potential cause may be the improved absorption and internalization of AZA-LPO relative to free AZA in breast cancer cells. [49,55,56].

## 4. Conclusions

In the present research, AZA-LIPO formulations were developed and optimized using BBD and anticancer potentials were evaluated in MCF-7 breast cancer cells. The AZA-LIPO was prepared by a lipid thin film hydration technique. The selected CPPs were *w*/*v* lipid concentration (Y_1_), *w*/*v* cholesterol concentration (Y_2_) and sonication time (min) (Y_3_) with their respective levels −1 low, 0 medium, +1 high, whereas the dependent variables were particle size (nm) and % drug entrapment efficiency (%). The predicted independent variable was used to prepare formulation in a lab experimentally and predicted values of software for response were found to be very close to the experimental values i.e., predicted value was 110 nm and observed value was 107 nm; similarly, EE% was observed to be 85.2% in comparison to predicted 81.32%. The optimized AZA-LIPO formulation showed an acceptable particle size, homogenous size distribution, spherical shape, high entrapment efficiency and controlled drug release profile. The MTT cytotoxicity assay on MCF-7 cell line revealed AZA-LIPO was more cytotoxic compared to free AZA. Further, confocal microscopy results proved higher cellular uptake of AZA-LIPO than plain AZA in cells. The AZA-LIPO was more effective than free AZA in increasing caspase-3 and the pro-apoptotic Bax protein, while reducing the expression of the Bcl2 protein. Therefore, it can be concluded that proposed drug delivery system offers great prospects for anticancer therapy in future for breast cancer, only after establishing it with proper preclinical and clinical studies.

## Figures and Tables

**Table 1:** Independent variables with their responses and limits.

Actual Coded Value	A	B	C
−1	70	10	10
0	80	20	15
1	90	30	20
Y_1_	Lipid Concentration (%*w*/*v*)		
Y_2_	Cholesterol concentration (%*w*/*v*)		
Y_3_	Sonication Time (min)		
Dependent variables	Particle size (nm) and entrapment efficiency (%)		

**Table 2:** Effect of independent variable on various responses expressed as mean ± SD (n = 3).

Run	Y_1_	Y_2_	Y_3_	Particle Size (nm) ± SD	Entrapment Efficiency (%EE) ± SD
1	80	20	15	190 ± 2.12	69.8 ± 2.24
2	80	10	20	106 ± 3.23	76.7 ± 1.31
3	80	10	10	220 ± 4.11	65.1 ± 3.22
4	80	20	15	190 ± 1.14	70.5 ± 2.03
5	70	10	15	109 ± 2.02	83.1 ± 0.52
6	80	30	20	202 ± 5.17	66.2 ± 2.14
7	70	20	20	116 ± 2.24	76.2 ± 1.02
8	80	20	15	190 ± 1.45	71.3 ± 4.01
9	90	10	15	111 ± 3.26	60.3 ± 3.36
10	90	20	10	175 ± 4.02	56.3 ± 2.13
11	90	30	15	119 ± 2.31	62.4 ± 5.24
12	70	20	10	168 ± 5.12	74.8 ± 1.18
13	80	20	15	190 ± 6.31	72.8 ± 0.53
14	90	20	20	104 ± 8.01	69.7 ± 4.12
15	70	30	15	107 ± 1.17	85.2 ± 0.51
16	80	20	15	192 ± 2.38	73.1 ± 2.18
17	80	30	10	235 ± 3.54	75.9 ± 1.27
T1	70	30	13	115 ± 1.41	80.45 ± 2.46
T2	70	20	10	124 ± 2.84	79 ± 1.13

**Table 3:** Evaluation of design checks point for T1 and T2.

Response		Runs		
		T1	T2	Optimized Formulation
Particle size (nm)	Observed	115	124	107
	Predicted	130	145	110
	Residuals	7	8	−4.82
EE (%)	Observed	80.45	79	85.2
	Predicted	75.2	72.8	81.32

**Table 4:** Drug entrapment efficiency and drug loading of optimized batch.

Batch No.	Excipients Drug Ratio	Particle Size (nm) ± SD	PDI ± SD	%Entrapment Efficiency ± SD	%Drug Loading ± SD
B1	60:40:10	119 ± 5	0.07 ± 0.004	60.3% ± 2%	5.48% ± 1%
B2	70:30:10	107 ± 3	0.035 ± 0.002	85.2% ± 1%	5.82% ± 2%
B3	75:25:10	109 ± 6	0.082 ± 0.005	43.2% ± 3%	3.92% ± 4%
B4	85:15:10	190 ± 8	0.163 ± 0.004	29% ± 4%	2.64% ± 3%
B5	90:10:10	111 ± 2	0.049 ± 0.003	60% ± 1%	5.45% ± 1%

## Data Availability

Not Applicable.
